# Analysis of early phase and subsequent phase III stroke studies of neuroprotectants: outcomes and predictors for success

**DOI:** 10.1186/2040-7378-6-2

**Published:** 2014-02-07

**Authors:** Jens Minnerup, Heike Wersching, Matthias Schilling, Wolf Rüdiger Schäbitz

**Affiliations:** 1 Department of Neurology, University of Münster, Albert-Schweitzer-Campus 1, 48149 Münster, Germany; 2 Institute of Epidemiology and Social Medicine, University of Münster, Domagkstrasse 3, 48149 Münster, Germany; 3 Department of Neurology, Bethel, Burgsteig 13, 33617 Bielefeld, Germany

**Keywords:** Stroke, Neuroprotection, Clinical trial design, Prediction of trial results

## Abstract

**Background:**

Efficacy of neuroprotective treatments for ischemic stroke was not convincingly demonstrated in clinical phase III trials so far, whereas some preceding early phase studies found neuroprotection to be beneficial. We aimed to determine the frequency with which phase III studies are preceded by positive early phase studies, and to identify characteristics of early phase studies that are associated with correct prediction of phase III studies.

**Methods:**

We identified phase III studies and corresponding early phase studies of neuroprotective treatments for stroke. Data on study characteristics of early phase trials were extracted and compared between studies that were classified according to their results as “false positive” and “true neutral” using logistic regression analysis.

**Results:**

Forty-six phase III studies and 59 corresponding early phase studies were identified. Only one phase III study was positive and this study was followed by a larger negative study. Twenty-two (37.3%) early phase studies were considered to be false positive and 37 (62.7%) to be true neutral. None of the early phase study characteristics were significantly associated with correct prediction of phase III studies.

**Conclusions:**

More than one third of early phase studies on neuroprotective stroke treatments are false positive. Neither the results nor specific study design characteristics of early phase stroke studies reliably predict success in phase III trials. Further efforts are needed to improve early phase studies regarding its predictability and to identify those early studies that should be advanced to phase III trials.

## Background

Neuroprotective treatments for stroke were considered to be promising for clinical development. This process usually progresses from phase I to phase II to phase III studies. The primary objectives of phase I studies are to assess the safety and tolerability of a treatment in a small group of healthy participants or patients. Some phase I studies also intend to gain early evidence of effectiveness [1]. Phase II trials evaluate the efficacy of a drug and further investigate its safety in a larger group of patients. In phase III trials a therapeutic intervention is compared to standard treatment to confirm its efficacy. Phase III studies are usually required for approval by regulatory agencies and for adoption of new therapies. Hundreds to thousands of patients are enrolled in phase III studies making these time consuming and expensive. So far, numerous neuroprotection phase III trials for ischemic stroke have been completed, none of which demonstrated unequivocal efficacy of the investigated treatment [2]. In contrast, some preceding early phase (phase I and II) studies found neuroprotective treatments to be beneficial regarding clinical outcome.

We aimed to determine the frequency with that phase III studies are preceded by positive early studies, and to identify study characteristics of early phase studies that are associated with correct prediction of subsequent phase III studies. Determination of these study characteristics can help to improve the trial design of future phase I and II stroke studies. Moreover, it might allow to evaluate the results of existing early phase studies regarding the capability for successful progression to phase III.

## Methods

### Identification and data extraction of phase III studies

For identification of phase III clinical trials on neuroprotective treatments for acute ischemic stroke we systematically searched the databases *Clinicaltrials.gov* (searched in November 2012) and *The Internet Stroke Center* (searched in November 2012) [1,3]. The search strategy for *Clinicaltrials.gov* used the terms “Closed Studies” for the search term ‘*Recruitment’*, “All studies” for the search term ‘*Study Result’*, “Interventional Studies” for the search term ‘*Study Type’*, and “Phase III” for the search term ‘*Phase’*. For search in *The Internet Stroke Center* we used the terms “Completed” and “Terminated” for the search term ‘*Status*’ and the term “Phase 3” for the search term ‘*Phase*’. In addition, review articles on clinical acute stroke studies were reviewed for phase III trials [2,4-6]. To include only studies on acute stroke, the search was limited to those studies with treatment initiation within 72 hours after stroke onset. Only studies on neuroprotection were included. Studies of thrombolytic, antithrombotic, or antihypertensive agents without neuroprotective properties were excluded. Publications of thus identified phase III studies were retrieved and data were extracted. Only articles in English were included. When studies were not published in full data were obtained from the *Internet Stroke Center* or from Cochrane Stroke Group reviews. Results of phase III studies were judged to be either “positive”, “neutral” or “negative”. A study was defined to be “positive” if the primary end points were reached or if the neuroprotective treatment was superior to the placebo treatment regarding functional recovery or mortality in cases the primary end point was not stated. Additional data that were retrieved include the maximum time of treatment initiation after symptom onset, the dose and route of administration of the neuroprotective treatment, and the number of patients included in the study.

### Identification, selection, and data extraction of early phase studies

Phase I and phase II studies of neuroprotective therapies of identified phase III studies were searched using the database Pubmed (searched at November 2012). This strategy included the words “stroke” or “ischemia” or “infarct” AND “drug name” or “abbreviated drug name”. In addition, articles of phase III studies were searched for preceding phase I and phase II studies. Except the terms “Phase I” and “Phase II” for the search term ‘*Phase’* the same search strategy as described for phase III studies was used for searching the databases *Clinicaltrials.gov* (searched in November 2012) and *The Internet Stroke Center* (searched in November 2012). Only articles in English were included. In the absence of full publication abstracts were analyzed when all required data were available by the Internet Stroke Center or from Cochrane Stroke Group reviews. Studies were determined as “positive” or “neutral” or “negative” regarding treatment efficacy as judged by the authors in the publication [7]. Moreover, data on the following characteristics of phase I and phase II studies were extracted: Number of patients enrolled, trial setting (single-center or multicenter), randomization, blinded outcome assessment, industry sponsoring, dose–response investigation, time point of outcome assessment, use of imaging endpoints (e.g. infarct size), and use of the same therapeutical time window, the same dose and the same route of administration as in the corresponding phase III trial. The selection of considered study characteristics was based on previously published articles on the design of acute stroke studies [7,8].

### Statistical analysis

Early phase studies (phase I and II) were assigned to their corresponding phase III trial. As early clinical stroke studies were frequently not clearly specified as phase I or phase II studies we subsumed these in one category. For treatments of which more than one phase III trials exists, phase I and phase II studies were allocated to their immediately following phase III studies. Phase I and II studies were classified as “true neutral” or “false positive”. Those with positive results and subsequent negative phase III studies were classified as “false positive” and those with neutral results and subsequent negative phase III studies were classified as “true neutral”. No phase I or phase II studies with negative results could be identified. As conflicting results of phase III studies on the efficacy of NXY-059 exist, the larger (neutral) Stroke-Acute Ischemic NXY Treatment Trial (SAINT) II trial was used as reference for the classification of phase I and phase II studies on NXY-059. For comparison of study characteristics between “false positive” and “true neutral” phase I and II studies we applied unadjusted (crude) logistic regression analysis and a multivariable model that included all study characteristics simultaneously. The level of significance was defined as a two-tailed P < 0.05. The analyses were carried out using SAS 9.2 and the Statistical Package of Social Sciences (version 21).

## Results

### Identified phase III studies and their results

The search strategy yielded 153 studies by searching the database *Clinicaltrials.gov* and 165 studies by searching the database *The Internet Stroke Center*. Seven further studies were identified by searching review articles on clinical acute stroke studies. Of the identified studies 46 phase III studies fulfilled the inclusion criteria (Table 1). In these studies 34 different neuroprotective treatments were investigated. The studies were published between 1988 and 2012. Four studies were not published (POST-010 and POST-011, Eliprodil Phase III, Fosphenytoin Phase III, and ARTIST+) and information on these studies was obtained from the Internet Stroke Center database. The median number of patients enrolled in the included studies was 670.5 (range 126 to 3306). Forty-three studies were neutral (active treatment and control not significantly different), two studies were negative (control superior to active treatment), and only one study was positive (active treatment superior to control). However, the only positive study (SAINT I) was followed by a larger negative study (SAINT II).

**Table 1:** Identified phase III studies

**Study acronym/study title**	**Year of publication**	**Intervention**	**No. of subjects**	**Result**
PAIS	2009	Acetaminophen	1400	Neutral
AHAIS	2001	Aptiganel	628	Neutral
BEST	1988	Atenolol, Propanolol	302	Neutral
POST-010 and POST-011	*	BMS-204352	1978	Neutral
SCAST	2011	Candesartan	2004	Neutral
Cervene phase 3	2000	Cervene (Nalmefene)	368	Neutral
Citicoline ECCO 2000	2001	Citicoline	899	Neutral
Citicoline 007	1999	Citicoline	394	Neutral
ICTUS	2012	Citicoline	2298	Neutral
CLASS	1999	Clomethiazole	1360	Neutral
CLASS-I	2002	Clomethiazole	1198	Neutral
EGASIS	2006	Diazepam	880	Neutral
MACSI	2013	DP-b99	446	Neutral
EAIS	1998	Ebselen	302	Neutral
EAST	2009	Edaravone	814	Neutral
Eliprodil phase III	*	Eliprodil	483	Neutral
EAST	2001	Enlimomab	625	negative
ESS	2009	Epoetin Alfa	522	Neutral
Fiblast phase III	2002	Fibroblast growth factor	286	Neutral
FIST	1996	Flunarizine	331	Neutral
Fosphenytoin phase III	*	Fosphenytoin	462	Neutral
GAIN International	2000	Gavestinel (GV150526)	1804	Neutral
GAIN Americas	2001	Gavestinel (GV150526)	1367	Neutral
EST	1994	GM1 ganglioside	792	Neutral
SASS	1994	GM1 ganglioside	287	Neutral
IASSH	1989	GM1 ganglioside	502	Neutral
ASCLEPIOS	1994	Israpidine	357	Neutral
LUB-INT-13	2000	Lubeluzole	1786	Neutral
Lub	1997	Lubeluzole	721	Neutral
IMAGES	2004	Magnesium	2589	Neutral
PRISTINE	1996	Naftidrofuryl	620	Neutral
ANS	1992	Nimodipine	1064	Neutral
INWEST	1994	Nimodipine	295	negative
TRUST	1990	Nimodipine	1215	Neutral
VENUS	2001	Nimodipine	454	Neutral
SAINT II	2007	NXY-059	3306	Neutral
SAINT I	2006	NXY-059	1722	positive
RREACT	2007	ONO-2506, Arundic acid	841	Neutral
PASS	1997	Piracetam	927	Neutral
mRECT	2009	Repinotan	681	Neutral
ASSIST	2000	Selfotel (CGS19755)	567	Neutral
RANTTAS	1996	Tirilazad mesylate	556	Neutral
RANTTAS II	1998	Tirilazad mesylate	126	Neutral
NEST-2	2009	Transcranial laser therapy	660	Neutral
ASTIN	2003	UK279,276	966	Neutral
ARTIST+	*	YM872	312	Neutral

*Study was not published, information on study was obtained from the Internet Stroke Center.

### Early phase study characteristics and their association with phase III results

Fifty-nine early phase studies, investigating 26 neuroprotective treatments were identified. The characteristics of these studies are shown in Table 2. For 8 treatments (combination of Atenolol and Propanolol, BMS-204352, Diazepam, Eliprodil, Enlimomab, Fibroblast Growth Factor, Fosphenytoin, Israpidine) evaluated in phase III studies no preceding randomized phase I/II trials were identified. The median number of subjects enrolled in phase I/II studies was 92 (range 25 to 725). More than half (61.0%) of the studies were sponsored by industry. The majority of studies were multicenter studies (69.5%). Randomization was reported in 57 (96.6%) and blinded outcome assessment in 52 (88.1%) of the included phase I/II studies. In 16 (27.1%) studies the dose–response relationship was explored and in 15 (25.4%) studies an imaging endpoint in addition to clinical endpoints was used. The median duration of follow-up for endpoints was 87 days (range 3 to 365). Compared to corresponding phase III trials in phase I and phase II studies the same therapeutical time window, the same dose, and the same route of administration was used in 18 (32.2%), 29 (49.2%), and 50 (84.7%), respectively.

**Table 2:** Characteristics of early phase studies and associations with phase III study results

**Study characteristics**	**All early phase studies (n = 59)**	**True neutral (n = 37)**	**False positive (n = 22)**	**aOR [95%-CI]**	**P**
No. of subjects, median (IQR)	92.0 (46.0-176.0)	100.0 (46.0-157.0)	91.5 (49.3-197.5)	1.00 [0.99-1.00]	0.713
Industry sponsored, n (%)	36 (61.0)	25 (67.6)	11 (50.0)	1.73 [0.40-7.54]	0.465
Multicentered, n (%)	41 (69.5)	28 (75.7)	13 (59.1)	1.48 [0.28-7.87]	0.643
Randomized, n (%)	57 (96.6)	35 (94.6)	22 (100.0)	0.00 [0.00-∞]	1.000
Blinded outcome assessment, n (%)	52 (88.1)	34 (91.9)	18 (81.8)	3.95 [0.26-60.30]	0.323
Dose–response investigated, n (%)	16 (27.1)	12 (32.4)	4 (18.2)	1.25 [0.26-6.07]	0.786
Use of imaging endpoint, n (%)	15 (25.4)	8 (21.6)	7 (31.8)	0.46 [0.12-1.81]	0.263
Duration of follow-up for endpoints*, median (IQR), days	87.0 (30.0-90.0)	90.0* (30.0-90.0)	41.0 (28.0-90.0)	1.00 [0.99-1.01]	0.722
Same characteristics in phase I/II studies as in subsequent phase III studies					
Same therapeutic time window, n (%)	19 (32.2)	10 (27.0)	9 (40.9)	0.69 [0.18-2.68]	0.588
Same dose, n (%)	29 (49.2)	18 (48.6)	11 (50.0)	0.94 [0.28-3.14]	0.916
Same route of administration, n (%)	50 (84.7)	34 (91.9)	16 (72.7)	3.33 [0.59-18.86]	0.174

IQR, interquartile range, aOR (adjusted odds ratio) for comparison of study characteristics between “false positive” and “true neutral” studies in a multivariable logistic regression analysis, CI, confidence interval, *one study did not report the exact duration of follow-up as endpoints were determined at discharge (mean length of hospital stay in this study was 9.5 days in the verum group and 11.2 days in the placebo group).

Twenty-two (37.2%) early phase studies were considered to be positive and 37 (62.7%) to be neutral. Characteristics of early phase studies and their associations with prediction of phase III study results are shown in Table 2. We found no study characteristic of early phase studies to be significantly associated with correct prediction of phase III results, neither in a univariate nor in multivariable logistic regression analysis.

In one study endpoints were determined at discharge and mean length of hospital stay was reported to be 9.5 days in verum treated patients and 11.2 days in the placebo group. Therefore duration to follow-up for endpoints in this study was estimated to be ten days (results of the regression model remained unchanged when duration to follow-up of this study was largely varied).

## Discussion

So far no phase III study convincingly demonstrated the efficacy of a neuroprotective treatment for acute ischemic stroke. In contrast more than one third of early phase studies included in our analysis reported neuroprotectants to be beneficial and thus yielded false positive results. We found no single characteristic of early phase studies to be significantly associated with correct prediction of phase III study results.

The question remains why promising results from early phase studies were not reproduced in phase III studies. The majority of analyzed early phase studies shared relevant features with phase III studies, such as randomization, blinded outcome assessment, and multicentricity. The main difference between early phase and phase III studies remains the number of enrolled patients, thus potentially being the reason for the discrepant results. This assumption is emphasized by results of a previous analysis of clinical stroke studies which found a decreased likelihood of a positive trial results with increasing sample size [7].

Different approaches were used to evaluate which early studies results can be considered as encouraging and should therefore be advanced to phase III. The method developed by Mandava and Kent is based on the assumption of an imbalance in randomization of baseline factors contributes to misleading results of early phase studies [4). In their model randomization errors are minimized by comparisons with an outcome function derived from a large number of pooled control arms. Using this model the failure of the SAINT II and of the Abciximab Emergent Stroke Treatment Trial (AbESTT) could have been predicted [4]. Although promising, this model has, however, not been evaluated prospectively so far.

The method used in our study was previously applied on cancer studies [9,10]. In an analysis of 351 early phase studies on targeted therapies for cancer multiple institution participation, industry sponsoring, and a shorter time period between publication of early phase and phase III studies were predictive factors for success in subsequent phase III trials [9]. In another study on chemotherapies for cancer treatment, however, none of the early phase study characteristics significantly predicted results of phase III studies [10]. In contrast to phase III studies on ischemic stroke a remarkable number of cancer phase III studies included in the analyses were positive. Hence the method to determine characteristics of phase II studies that predict success in subsequent studies cannot be simply adopted for stroke trials. We therefore slightly modified this approach and aimed to identify characteristics that are associated with “false positive” and “true neutral” results.

A limitation of our analysis might be bias caused by unpublished studies [5,7]. However, to reduce the impact of publication bias on our results we also included studies that were not published in full and obtained data from the *Internet Stroke Center* and from Cochrane Stroke Group reviews. A further limitation of our analysis is the fact that early phase studies are usually not powered to detect differences of the clinical outcome. However, the results on efficacy in early studies are frequently the basis for testing in phase III trials.

## Conclusion

Our study shows that more than one third of early phase studies on neuroprotective treatments for stroke are false positive. We found no single early phase study characteristic whose presence or absence reliable predicts success in phase III trials. Further efforts are needed to improve early phase stroke studies regarding its predictability and to identify those early studies that should be advanced to phase III trials.

## Competing interests

Wolf-Rüdiger Schäbitz received honoraria for several presentations on citicoline from Trommsdorff, Jens Minnerup has received funding from Trommsdorff.

## Authors’ contributions

JM: Study concept, analysis and interpretation of data, acquisition of data, statistical analysis. HW: Study concept, analysis and interpretation of data, statistical analysis. MS: Study concept, analysis and interpretation of data. WS: Study concept, study supervision. All authors read and approve the manuscript.

## Authors’ information

Matthias Schilling and Wolf Rüdiger Schäbitz are senior-authorship.
